# Identifying Prognostic Markers From Clinical, Radiomics, and Deep Learning Imaging Features for Gastric Cancer Survival Prediction

**DOI:** 10.3389/fonc.2021.725889

**Published:** 2022-02-02

**Authors:** Degan Hao, Qiong Li, Qiu-Xia Feng, Liang Qi, Xi-Sheng Liu, Dooman Arefan, Yu-Dong Zhang, Shandong Wu

**Affiliations:** ^1^ Intelligent Systems Program, University of Pittsburgh, Pittsburgh, PA, United States; ^2^ Department of Radiology, The First Affiliated Hospital with Nanjing Medical University, Nanjing, China; ^3^ Department of Radiology, University of Pittsburgh, Pittsburgh, PA, United States; ^4^ Department of Biomedical Informatics, University of Pittsburgh, Pittsburgh, PA, United States; ^5^ Department of Bioengineering, University of Pittsburgh, Pittsburgh, PA, United States

**Keywords:** gastric cancer, survival analysis (source: MeSH NLM), multi-modal data analysis, radiomics, deep learning - CNN

## Abstract

**Background:**

Gastric cancer is one of the leading causes of cancer death in the world. Improving gastric cancer survival prediction can enhance patient prognostication and treatment planning.

**Methods:**

In this study, we performed gastric cancer survival prediction using machine learning and multi-modal data of 1061 patients, including 743 for model learning and 318 independent patients for evaluation. A Cox proportional-hazard model was trained to integrate clinical variables and CT imaging features (extracted by radiomics and deep learning) for overall and progression-free survival prediction. We further analyzed the prediction effects of clinical, radiomics, and deep learning features. Concordance index (c-index) was used as the model performance metric, and the predictive effects of multi-modal features were measured by hazard ratios (HRs) at pre- and post-operative settings.

**Results:**

Among 318 patients in the independent testing group, the hazard predicted by Cox from multi-modal features is associated with their survival. The highest c-index was 0.783 (95% CI, 0.782-0.783) and 0.770 (95% CI, 0.769-0.771) for overall and progression-free survival prediction, respectively. The post-operative variables are significantly (p<0.001) more predictive than the pre-operative variables. Pathological tumor stage (HR=1.336 [overall survival]/1.768 [progression-free survival], p<0.005), pathological lymph node stage (HR=1.665/1.433, p<0.005), carcinoembryonic antigen (CEA) (HR=1.632/1.522, p=0.02), chemotherapy treatment (HR=0.254/0.287, p<0.005), radiomics signature [HR=1.540/1.310, p<0.005], and deep learning signature [HR=1.950/1.420, p<0.005]) are significant survival predictors.

**Conclusion:**

Our study showed that CT radiomics and deep learning imaging features are significant pre-operative predictors, providing additional prognostic information to the pathological staging markers. Lower CEA levels and chemotherapy treatments also increase survival chances. These findings can enhance gastric cancer patient prognostication and inform treatment planning.

## Introduction

Gastric cancer is one of the leading causes of death worldwide (1). Accurate survival prediction of gastric cancer patients can inform clinical decision making and benefit treatment planning (2). Since 1977, the American Joint Committee on Cancer (AJCC) staging system is the guideline for treatment allocation and prognostic prediction on gastric cancer patients (3–5). However, the staging system is hard to account for the large variations in survival outcomes.

Previous studies have reported a variety of clinical factors indicative of gastric cancer prognosis, including serum tumor markers, lymphovascular invasion, perineural invasion, histological grade, etc. (6–10). Recent studies also showed that quantitative imaging features, such as radiomics and deep learning modeling, are associated with survival/prognosis of gastric cancer patients (11, 12). Radiomics represent predefined quantitative imaging descriptors. Deep learning (13) can automatically extract imaging features from high-dimensional imaging data, but these features are less intuitive than radiomics descriptors.

It is expected that the combination of multi-modal data, such as demographic information, clinical variables, imaging data, histopathologic findings, lab measurements, therapeutic interventions, can empower survival analysis of gastric cancer (14). Currently, it lacks understanding of the interaction and relationship of the multi-modal features for predicting gastric cancer survival. The purpose of this study is to integrate clinical variables, radiomics features, and convolutional neural network (CNN)-identified deep learning features to predict overall and progression-free survival on gastric cancer patients and identify key prognostic markers from the multi-modal data modeling at pre- and post-operative settings.

## Materials and Methods

### Overview

**Figure 1:**
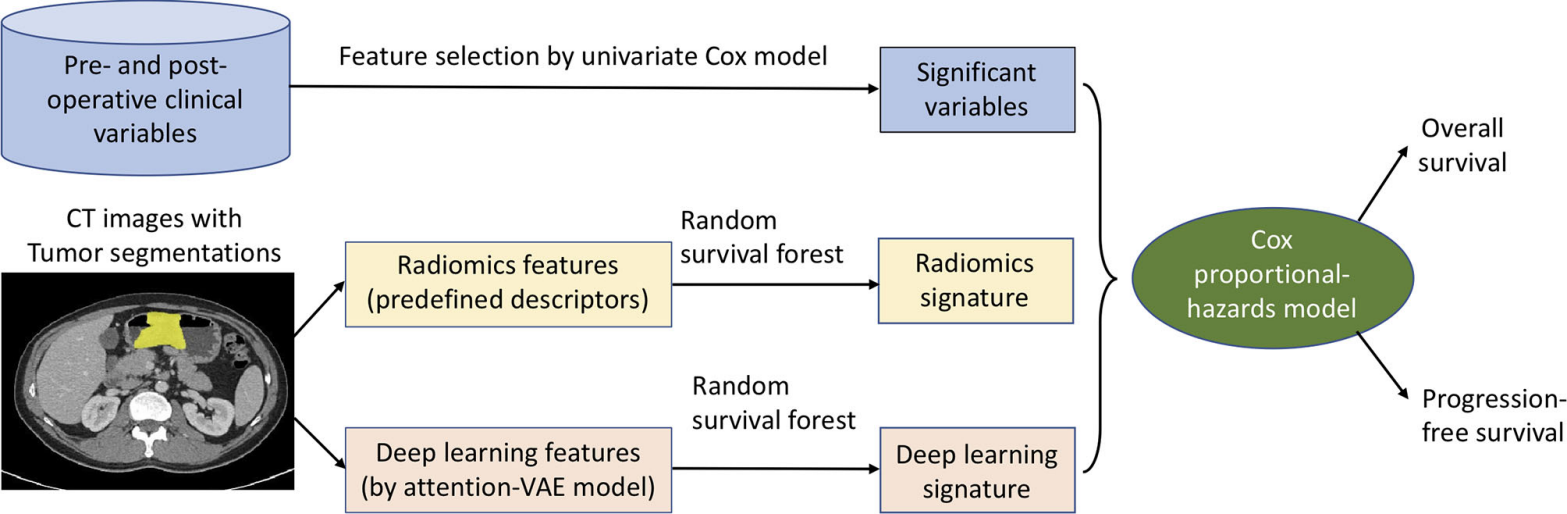
Machine learning of multi-modal features for gastric cancer survival prediction and interpretation. The significant clinical variables, radiomics signature, and deep learning signature were integrated in the Cox model for survival prediction, and the effects of these features were measured and analyzed by hazard ratios at pre- and post-operative settings.

We built a machine learning prognostic model (**Figure 1**) for overall and progression-free survival prediction after gastrectomy, by integrating multi-modal data: clinical variables (including demographic information, lab tests, pathology, and treatment data), intra-tumor radiomics, and deep learning features of the tumor regions. The large set of radiomics (or deep learning) features were aggregated to generate a signature by the random survival forest method (15). We used the classic Cox proportional-hazards (Cox in short) model for data integration, survival prediction, and effect measurement.

### Study Cohort

We performed a retrospective study that received approvals by a local ethics committee and an institutional review board with a waiver of written informed consent. Our study complies with the 1964 Helsinki declaration and its later amendments. Initially a total of 1,647 patients with pathologically confirmed gastric cancer during 2014 to 2018 were identified for the study. The exclusion criteria included: i) patients who failed to undergo radical surgery; ii) patients with diagnosis of other cancers in addition to gastric cancer; iii) patients with any intervention or therapy before surgery; iv) patients with poor imaging quality unacceptable for computational analysis; and v) patients without pre-operative CT imaging available. Finally, 1,061 patients were included for analysis, which were randomly split to two independent study groups: Group-A of 743 patients (70%) for model development and Group-B of 318 patients (30%) for independent evaluation. Patients were followed up every 3-6 months, starting from the time of gastrectomy and censored at the last alive contact or by the time of this study (i.e., 30 June 2019). For each patient, we collected various clinical data and a pre-operative contrast-enhanced computed tomography (CECT) scan.

### Clinical Variables

We collected a set of clinical data acquired before and after the gastrectomy operation. The pre-operative variables include lab tests [e.g., serum carcinoembryonic antigen (CEA) and carbohydrate antigen 19-9 (CA19-9)], demographic variables, qualitative radiologic staging variables [e.g., tumor depth invasion (rT) and lymph node invasion (rN)], tumor location assessed by radiographic imaging and endoscopy, histologic grades by endoscopic biopsy. The post-operative data includes chemotherapy treatment information as well as surgical pathology variables [e.g., pathologic tumor staging (pT), pathologic lymph node staging (pN), Lauren classification, gross appearance, surgical histologic grade, lymphovascular invasion (LVI), perineural invasion (PNI)]. See **Supplementary** for more details on the variable measurement. We performed univariate statistical tests for each variable (chi-squared test for discrete variables and Mann-Whitney U test for continuous variables) between Group-A and Group-B to measure their properties. In order to select variables that are substantially related to survival, univariate Cox analysis (16) was performed and those with a p-value < 0.10 were selected for subsequent joint modeling with imaging data.

### Radiomics Features Extracted From 3D Intra-Tumor Volume

Quantitative radiomic features are extracted from the segmented 3D tumor volume in the CECT images. The gastric tumor was segmented slice-by-slice and semi-automatically by two radiologists (QL and QXF) using an in-house developed and validated software (ONCO IMAG ANLY v 2.0; Shanghai Key Laboratory of MRI, ECNU, Shanghai, China). QL first segmented the lesion for all cases; and one week later, QL repeated segmentation on 30 patients to evaluate intra-reader variability. To evaluate inter-observer variability, QXF performed lesion segmentation on a selected subset of 30 patients. The lesion segmentation was conducted over approximately two months. A total of 1,210 radiomic features, which describe the tumor characteristics in terms of intensity, shape, texture, etc., are extracted from the segmented gastric tumor volumes using an open-source Python package Pyradiomics (17). The robustness of each radiomic feature between readers is measured using intra-class correlation coefficient (ICC).

### Deep Learning Features Extracted From the Full Images Focused on the Tumor Regions

**Figure 2:**
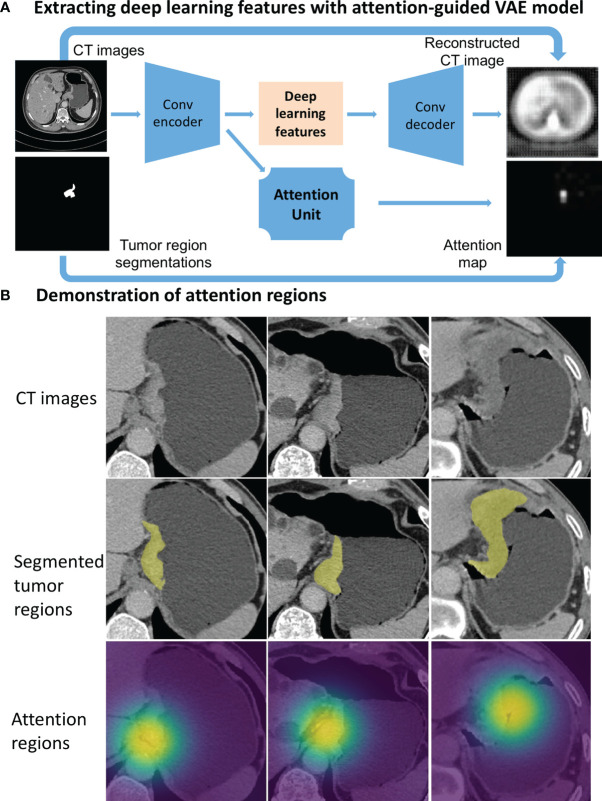
Deep learning feature extraction from CT images through an attention-guided Variational AutoEncoder (attention-guided VAE) model. **(A)** model structure. **(B)** Gastric tumor region (yellow annotations) and the attention regions (highlighted by heatmaps) identified by the attention-guided VAE model.

Deep learning was used to extract potentially different features from the approximate local regions around segmented tumor. To this end, we designed an attention-guided Variational AutoEncoder (attention-guided VAE) model (**Figure 2A**) to guide the feature learning. The model was trained with the manually segmented gastric tumor masks, where an attention unit was incorporated to learn an attention map around the segmented tumor regions. At the bottleneck of this model, the hidden layer outputs a 100-dimensional vector as the deep learning features to characterize the attended tumor regions. **Figure 2B** shows several examples of the attention regions identified by the deep learning model.

### Generating Aggregated Imaging Signatures by Random Survival Forest

Due to the relatively large number of radiomics features and deep learning features, direct use of the full set of features may result in overfitting in the Cox model. We employed random survival forest (15) to first select a substantially smaller subset from the 1,210 radiomic features, and from the 100 attention-VAE features, respectively. Random survival forest is an ensemble tree method that identifies a subset of outcome-correlated features based on their permutation feature importance (18). The random survival forest process produces a score indicating the survival probability and the score represents an aggregated signature of its selected features, from which we generated the radiomics signature and deep learning signature. The training of random survival forest models was performed on Group-A only and separately for the overall and progression-free survival prediction.

### Evaluation and Statistical Analysis

We evaluated and compared the survival prediction effects at 4 different settings, including using pre-operative data and post-operative data, separately (Setting 1), combination of the full set of pre- and post-operative data (Setting 2), and combination of only the variables that are shown in Setting 1 to be statistically significant (p<0.05) (Setting 3). In addition, we performed one more round of feature selection using the random survival forest method from the full set of data at Setting 1 and only the selected variables were combined for modeling (Setting 4).

In the deep learning feature extraction, the 743 patients in Group-A were randomly split into a training set (669 patients) and a validation set (74 patients) for model learning. The axial view CECT image with the largest cross-sectional area of tumor was selected as the input of the attention-VAE model. We used open-source software libraries PyTorch (19) to implement deep learning modeling, and scikit-survival (20) to implement random survival forest.

The model performance was measured on the independent Group-B of 318 patients using concordance index (c-index) (21). Hazard ratios were calculated to measure the effect of each individual variable/feature. In order to measure the effects more robustly, we repeated each experiment 20 times and calculated the average c-index values. We reported 95% confidence intervals of the c-index values using the non-parametric bootstrap method (22). We also conducted statistical comparisons on the model performance among Settings 1 to 4 using two-tailed Student’s t-test. We performed all statistical analyses using the R software (version 3.6.1, R Project for Statistical Computing) and Python (version 3.6.8). A two-sided p value less than 0.05 is considered statistically significant.

## Results

### Patient Characteristics

**Table 1** summarizes key characteristics of the study cohort in terms of 16 clinical variables. There are 8 pre-operative and 8 post-operative variables. The percentage of the average follow-up time is 23.6 months (range 1- 65 months). The median age is 61.7 ± 10.3 years. There are 762 male patients and 299 female patients. The time interval between the CECT examination and standard gastrectomy had a median of 9 days, ranging from 6 to 14 days. Between Group-A and Group-B, all the clinical variables are statistically similar (as shown in **Table 1**, all the p values are greater than or equal to 0.05). In Group-A, 355 (48%) patients underwent total gastrectomy while 388 (52%) patients underwent subtotal gastrectomy. In Group-B, the corresponding number was 163 (51%) and 155 (49%), respectively, for total and subtotal gastrectomy. In our cohort, there were 308 patients who did not undergo chemotherapy while they were eligible according to the National Comprehensive Cancer Network (NCCN) guideline on indications for chemotherapy (23), and there were 8 patients who underwent chemotherapy while they are ineligible per the NCCN guideline (23). The type of the chemotherapy varied across patients, including XELOX (oxaliplatin + capecitabine), SOX (S-1 + oxaliplatin), DS (docetaxel + S-1), etc. Our study cohort did not include patients who received neoadjuvant chemotherapy.

**Table 1:** Patient characteristics (i.e., 16 clinical variables) included for survival modeling.

Characteristic	Group-A for training (n = 743)	Group-B for independent test (n = 318)	p-value
**Preoperative variables**			
Age, mean ± Std	61.8 ± 9.7	62.0 ± 9.6	0.43
Sex, No. (%)			0.20
Male	541 (72.8)	221 (69.5)	
Female	202 (27.2)	97 (30.5)	
CA19-9 < 39 units/milliliter, No. (%)			0.27
Yes	94 (12.7)	35 (11.0)	
No	649 (87.3)	283 (89.0)	
CEA < 4.7 nanograms/milliliter, No. (%)			0.24
Yes	160 (21.5)	63 (19.8)	
No	583 (78.5)	255 (80.2)	
Biopsy histologic grade, No. (%)			0.63
Well/moderate	440 (59.2)	194 (61.0)	
Poor/undifferentiated	303 (40.8)	124 (39.0)	
Location, No. (%)			0.99
Upper	165 (22.2)	87 (27.4)	
Middle	232 (31.2)	96 (30.2)	
Lower	332 (44.7)	128 (40.3)	
Entire	14 (1.9)	7 (2.2)	
Radiologic T stage, No. (%)			0.05
rT1 stage	152 (20.5)	46 (14.5)	
rT2 stage	123 (16.6)	70 (22.0)	
rT3 stage	286 (38.5)	122 (38.4)	
rT4 stage	182 (24.5)	80 (25.2)	
Radiologic N stage, No. (%)			0.83
rN0 stage	281 (37.8)	113 (35.5)	
rN1 stage	196 (26.4)	81 (25.5)	
rN2 stage	134 (18.0)	65 (20.4)	
rN3 stage	70 (9.4)	34 (10.7)	
rN4 stage	62 (8.3)	25 (7.9)	
**Post-operative variables**			
Pathological T stage^†^, No. (%)			0.99
pT1 stage	202 (27.2)	86 (27.0)	
pT2 stage	95 (12.8)	39 (12.3)	
pT3 stage	202 (27.2)	85 (26.7)	
pT4 stage	244 (32.8)	108 (34.0)	
Pathological N stage^†^, No. (%)			0.69
pN0 stage	285 (38.4)	115 (36.2)	
pN1 stage	102 (13.7)	51 (16.0)	
pN2 stage	120 (16.2)	47 (14.8)	
pN3a stage	131 (17.6)	53 (16.7)	
pN3b stage	105 (14.1)	52 (16.4)	
Surgical histologic grade, No. (%)			0.44
Well/moderate	418 (56.3)	170 (53.5)	
Poor/undifferentiated	325 (43.7)	148 (46.5)	
Lauren classification, No. (%)			0.63
Intestinal type	407 (54.8)	180 (56.6)	
Diffuse/mixed type	336 (45.2)	138 (43.4)	
Gross appearance, No. (%)			0.75
Borrmann type I-III	715 (96.2)	304 (95.6)	
Borrmann type IV	28 (3.8)	14 (4.4)	
Lymphovascular invasion, No. (%)			0.51
Negative	457 (61.5)	188 (59.1)	
Positive	286 (38.5)	130 (40.9)	
Perineural invasion, No. (%)			0.86
Negative	443 (59.6)	187 (58.8)	
Positive	300 (40.4)	131 (41.2)	
Chemotherapy therapy, No. (%)			0.55
Yes	334 (45.0)	150 (47.2)	
No	409 (55.0)	168 (52.8)	

^†^According to the eighth edition AJCC Cancer Staging Manual.

### Selected Significant Variables/Features

Out of the 16 variables listed in **Table 1**, the following key variables were selected for modeling: 5 pre-operative variables (CEA, CA19-9, biopsy findings, rT, rN) and 7 post-operative variables (pT, pN, LVI, PNI, gross appearance, surgical histologic grade, and chemotherapy treatment). For the radiomics feature extraction, the average intra-observer ICC was 0.96 and the average inter-observer ICC was 0.86, indicating a good reliability. The most relevant radiomics features selected by random survival forest to generate the radiomics signatures are listed in **Table 2**, along with their respective ICC values.

**Table 2:** Radiomic features selected by random survival forest to generate the radiomics signatures for overall survival and progression-free survival.

Prediction	Radiomic feature name	Permutation importance	Intra-observer ICC	Inter-observer ICC
**Overall survival**	wavelet-HLL_firstorder_MeanAbsoluteDeviation	0.0068	0.999	0.999
	wavelet-HHH_glszm_SmallAreaLowGrayLevelEmphasis	0.0048	0.823	0.818
	log-sigma-5-0-mm-3D_glszm_SmallAreaHighGrayLevelEmphasis	0.0034	0.947	0.912
	original_shape_Maximum2DDiameterRow	0.0033	0.999	0.999
	original_glszm_ZoneVariance	0.0029	0.999	0.999
	wavelet-LLL_firstorder_Energy	0.0029	0.999	0.999
	original_shape_MajorAxis	0.0029	0.999	0.999
	wavelet-HLH_glszm_LargeAreaEmphasis	0.0028	0.987	0.977
	wavelet-HLH_glrlm_LongRunEmphasis	0.0027	0.996	0.994
	log-sigma-5-0-mm-3D_glszm_GrayLevelNonUniformity	0.0027	0.999	0.999
	wavelet-LHL_firstorder_MeanAbsoluteDeviation	0.0023	0.999	0.998
	original_shape_SurfaceVolumeRatio	0.0022	0.994	0.991
	original_glszm_SmallAreaEmphasis	0.0022	0.972	0.942
	original_firstorder_10Percentile	0.0021	0.996	0.992
	log-sigma-2-0-mm-3D_glszm_GrayLevelNonUniformity	0.0021	0.999	0.999
	wavelet-LLH_gldm_DependenceNonUniformity	0.0021	0.998	0.999
	wavelet-HLH_firstorder_Mean	0.002	0.984	0.962
	original_firstorder_Energy	0.002	0.999	0.999
	wavelet-HHL_glcm_ClusterTendency	0.0017	0.996	0.995
	wavelet-LLH_glrlm_GrayLevelNonUniformityNormalized	0.0017	0.998	0.996
**Progression-free survival**	original_glszm_SizeZoneNonUniformity	0.0037	0.999	0.999
	log-sigma-4-0-mm-3D_gldm_LargeDependenceHighGrayLevelEmphasis	0.0033	0.981	0.980
	wavelet-LLH_firstorder_RootMeanSquared	0.0032	0.999	0.999
	wavelet-LLH_gldm_LargeDependenceEmphasis	0.003	0.995	0.992
	original_shape_SurfaceArea	0.0029	0.999	0.999
	original_glszm_GrayLevelNonUniformity	0.0028	0.999	0.997
	log-sigma-5-0-mm-3D_glszm_LargeAreaHighGrayLevelEmphasis	0.0026	0.999	0.991
	wavelet-HHL_glcm_SumSquares	0.0025	0.999	0.999
	wavelet-HLL_glszm_ZoneVariance	0.0025	0.992	0.983
	wavelet-HHH_glszm_SmallAreaLowGrayLevelEmphasis	0.0024	0.823	0.818
	wavelet-HHH_glszm_SizeZoneNonUniformity	0.0023	0.929	0.929
	wavelet-HHL_glcm_JointEntropy	0.0022	0.999	0.999
	wavelet-LHH_firstorder_RobustMeanAbsoluteDeviation	0.002	0.999	0.999
	wavelet-LHL_glszm_LargeAreaLowGrayLevelEmphasis	0.002	0.996	0.944
	wavelet-HHL_glrlm_RunLengthNonUniformity	0.002	0.999	0.999
	wavelet-LLH_glszm_ZoneVariance	0.0018	0.998	0.997
	wavelet-HHH_glrlm_RunPercentage	0.0018	0.995	0.988
	original_firstorder_Variance	0.0018	0.995	0.991
	wavelet-HHH_glszm_SmallAreaEmphasis	0.0017	0.856	0.822
	wavelet-HHL_glszm_GrayLevelNonUniformityNormalized	0.0017	0.973	0.948

Larger permutation importance values indicate more important features for the prediction task. The intra-class correlation coefficient (ICC) values indicate reliability of the features.

### Performance of the Survival Prediction Models

**Table 3** shows the full survival prediction results with a comprehensive comparison under different settings. As can be seen at Setting 1, when only using the post-operative variables, the c-indexes are 0.783 for overall survival and 0.770 for progression-free survival. When only using the pre-operative variables, the corresponding c-indexes are 0.651 and 0.686, respectively. In both cases, the post-operative variables are significantly (p<0.001 for both overall and progression-free survival) more predictive than the pre-operative variables.

**Table 3:** Prediction performance of overall survival and progression-free survival and their comparisons at different settings.

Tasks	Variable groups	Variable names	Setting 1: Pre-operative / post-operative separate modeling			Setting 2: Combined modeling (full set of variabels in Setting 1)			Setting 3: Combined modeling (only significant variabels in Setting 1)			Setting 4: Combined modeling (applied feature selection to full set in Setting 1)		
			Hazard Ratio	p-value	C-index [95% CI]	Hazard Ratio	p-value	C-index [95% CI]	Hazard Ratio	p-value	C-index [95% CI]	Hazard Ratio	p-value	C-index [95% CI]
**Overall Survival**	Pre-operative variables	CEA	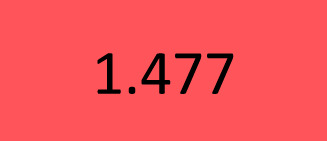	0.03	0.651 (0.649, 0.653)	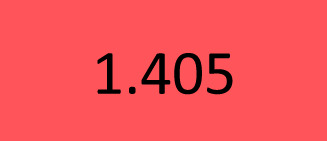	0.06	0.703 (0.702, 0.706)	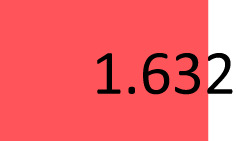	0.02	0.708 (0.706, 0.709)			0.721 (0.720, 0.722)
		CA199	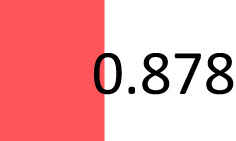	0.51		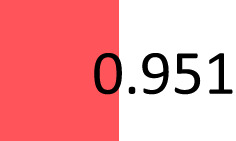	0.82							
		Biopsy histologic grade	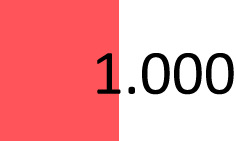	0.98		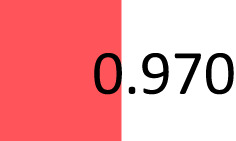	0.86							
		rT	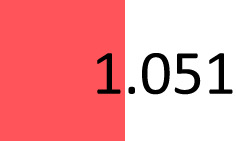	0.65		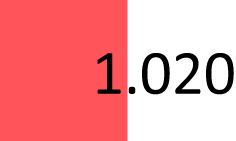	0.90					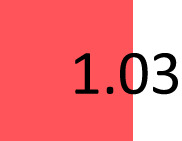	0.65	
		rN	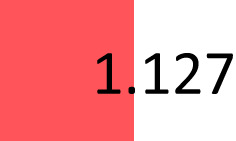	0.10		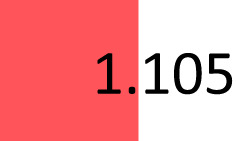	0.20					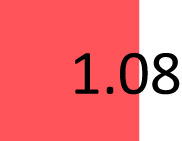	0.12	
		Deep learning signature	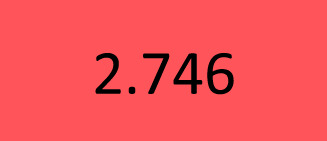	<0.005		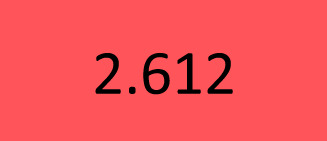	<0.005		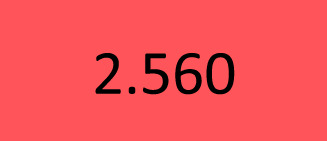	<0.005		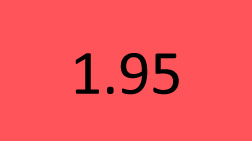	<0.005	
		Radiomics signature	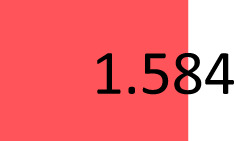	<0.005		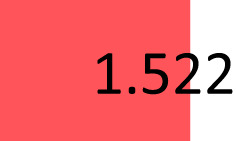	<0.005		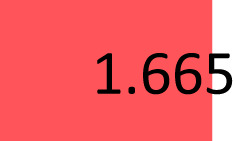	<0.005		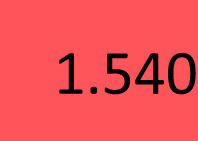	<0.005	
	Post-operative variables	pT	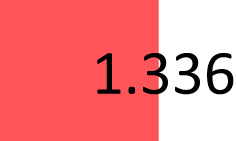	<0.005	0.783 (0.782, 0.783)	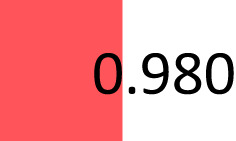	0.88		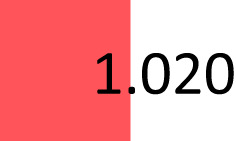	0.88		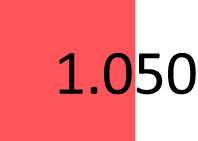	0.43	
		pN	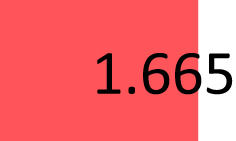	<0.005		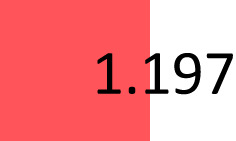	0.05		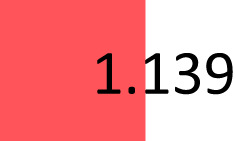	0.17		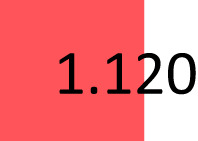	0.05	
		LVI	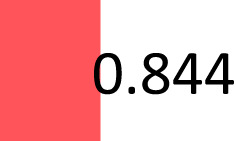	0.34		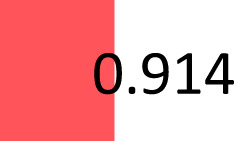	0.60							
		PNI	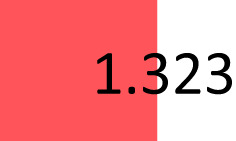	0.10		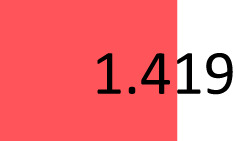	0.05							
		Gross appearance	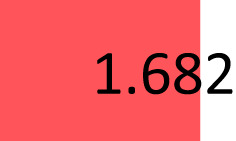	0.04		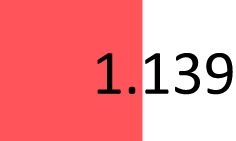	0.64		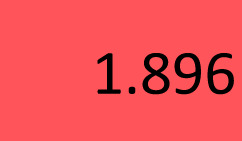	0.05				
		Surgical histologic grade	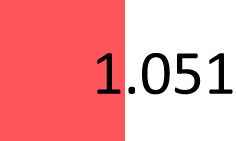	0.76		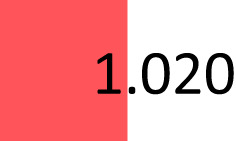	0.90					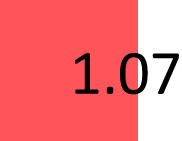	0.61	
		Chemotherapy	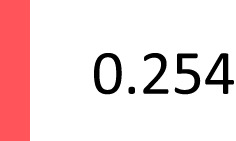	<0.005		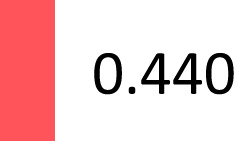	<0.005		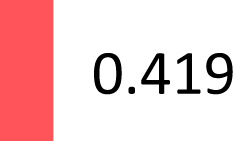	<0.005		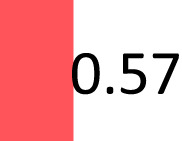	<0.005	
**Progression-Free Survival**	Pre-operative variables	CEA	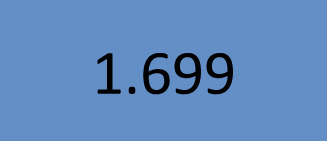	0.01	0.686 (0.685,0.687)	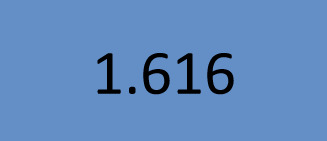	0.01	0.743 (0741,0.744)	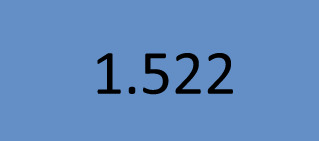	0.02	0.761 (0.759, 0.762)	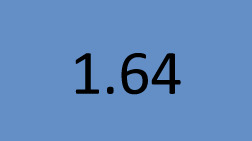	0.01	0.758 (0.757, 0.759)
		CA199	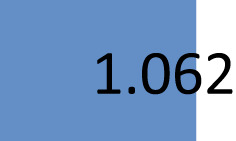	0.78		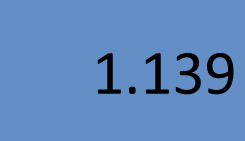	0.57							
		Biopsy histologic grade	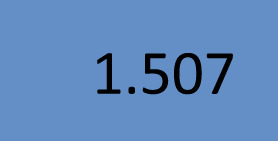	0.03		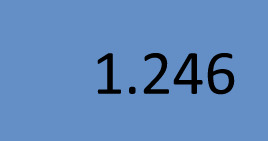	0.25					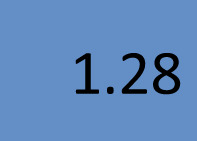	0.19	
		rT	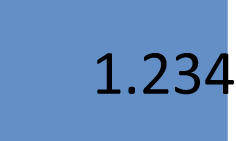	0.07		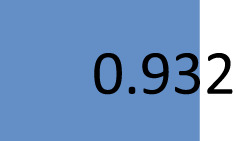	0.61					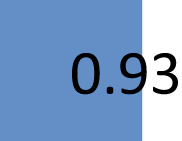	0.61	
		rN	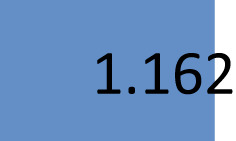	0.06		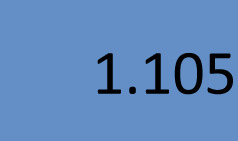	0.22					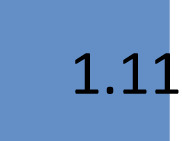	0.26	
		Deep learning signature	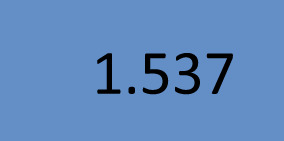	<0.005		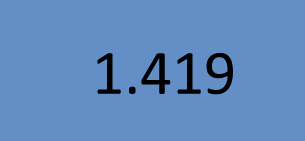	<0.005		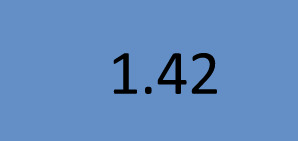	<0.005		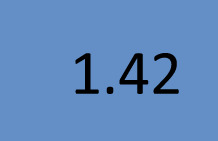	<0.005	
		Radiomics signature	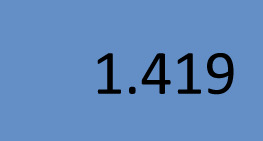	<0.005		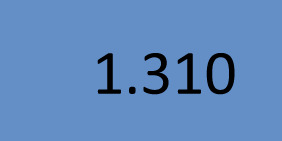	0.01		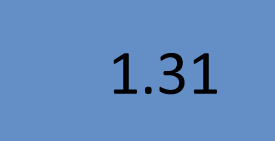	0.01		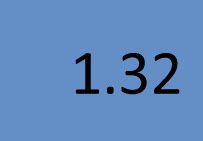	<0.005	
	Post-operative variables	pT	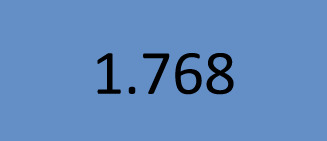	<0.005	0.770 (0.769, 0771)	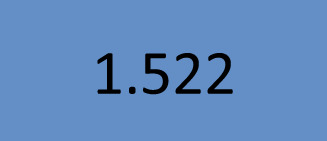	<0.005		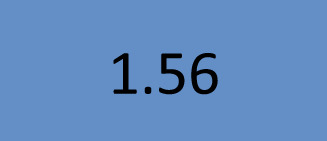	<0.005		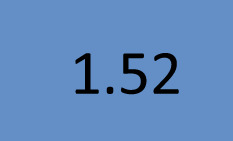	<0.005	
		pN	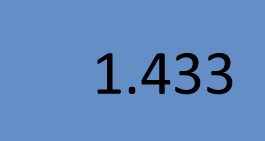	<0.005		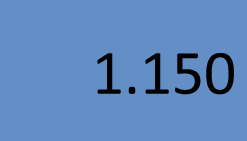	0.14		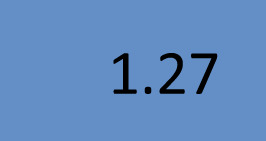	<0.005		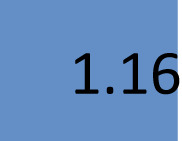	0.03	
		LVI	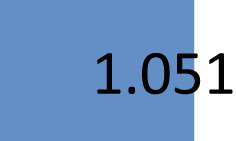	0.82		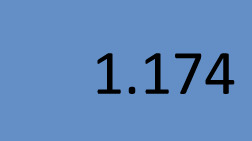	0.42							
		PNI	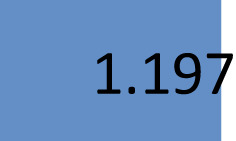	0.36		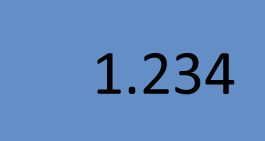	0.29							
		Gross appearance	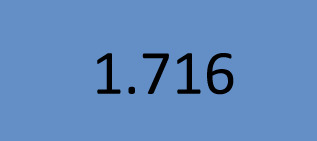	0.07		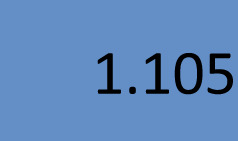	0.76		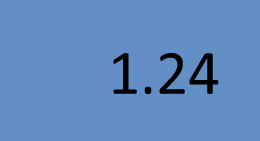	0.48				
		Surgical histologic grade	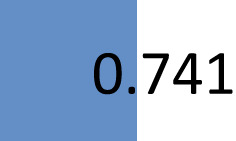	0.09		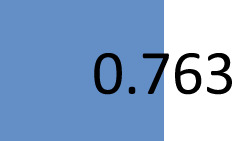	0.14							
		Chemotherapy	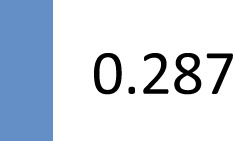	<0.005		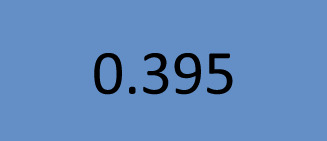	<0.005		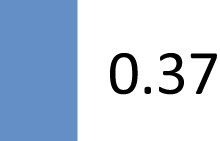	<0.005		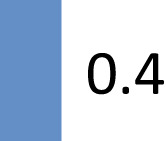	<0.005	

The length of the color bars in each cell represents the absolute value of the hazard ratios.

When the full set of the pre- and post-operative variables are combined (Setting 2), the respective c-index of overall and progression-free survival is 0.703 and 0.743, both outperforming (both p<0.001) the pre-operative variables alone but underperforming (both p<0.001) the post-operative variables alone. This implies that these variables may not be optimally integrated by the Cox model in Setting 2. When combining only the significant variables (those with p<0.05 at Setting 1), as shown at Setting 3, the c-index increases to 0.708 for overall survival prediction, slightly higher (p=0.19) than using all the variables (0.703) at Setting 2, while still significantly lower (p<0.001) than the post-operative variables (0.783) at Setting 1; meanwhile, the progression-free survival prediction shows a similar observation at Setting 3, where the c-index increases to 0.761, which is significantly higher (p<0.001) than using all the variables at Setting 2 (0.743), but again, significantly lower (p<0.001) than the post-operative variables (0.770) at Setting 1. At Setting 4, the c-index increases to 0.721 for overall survival prediction, which is still significantly (p<0.001) lower than the post-operative modeling (0.783) in Setting 1, but significantly (p<0.001) higher than the combined full set (0.703) at Setting 2. Likewise, at Setting 4, the performance pattern of the progression-free survival is similar to that of the overall survival. The comparisons of these results indicate the following: (I) when the full set of pre- and post-operative variables are all combined (Setting 2), the c-index values increase and become closer to, but are still lower than, just using the post-operative variables; and (II) regardless of using only the significant variables with p<0.05 (Setting 3) or using the variables selected by a second process of feature selection (Setting 4), the c-index is improved than using the full set at Setting 2. When comparing Setting 3 and Setting 4, the c-index for overall survival is higher (p<0.001) at Setting 4, while the c-index for progression-free survival is higher (p=0.02) at Setting 3. This indicates that the two methods of selecting subset variables for modeling (i.e., Settings 3 and 4) have respective advantages for the two different survival prediction tasks.

### Effect Evaluations of the Multi-Modal Features

After comparing the prediction model’s performance, here we analyze the prediction effects of individual variables in terms of their hazard ratios. Here we first look at the overall survival prediction. It shows CEA (HR=1.477; p=0.03), deep learning signature (HR=2.746; p<0.005), and radiomics signature (HR=1.584; p<0.005) are significant variables for pre-operative prediction; for post-operative prediction, the significant variables are pT (HR=1.336; p<0.005), pN (HR=1.665; p<0.005), gross appearance (HR=1.682, p=0.04), and chemotherapy (HR=0.254, p<0.005). At Setting 2, the two imaging variables (i.e., deep learning signature and radiomics signature) remain significant with similar hazard ratios, along with the following new observations: CEA became marginal (p=0.06), pT became insignificant (p=0.88)), PNI became marginally significant (p=0.05), gross appearance became insignificant (p=0.64), and chemotherapy’s hazard ratio increased to 0.440 from 0.254. At Setting 3, those significant variables still remain significant except the pT and pN; it should be noted that in this case, the c-index (0.708) is much lower than the post-operative prediction (0.783), indicating very likely that the effects of pT and pN were lost in this setting. It is interesting to see that at Setting 4, rT and rN are selected in the models; however, as their p values are greater than 0.05 and the HRs are close to 1, the predictive values of rT and rN are limited when combined with other more significant variables. Comparing Setting 3 and Setting 4, CEA and gross appearance are significant in Setting 3, but they are not selected at Setting 4; in contrast, pN is marginally significant (p=0.05) at Setting 4 but is in-significant (p=0.17) at Setting 3; the two imaging signatures and chemotherapy treatment remain the significant predictors at both Setting 3 and Setting 4 for the overall survival prediction.

Similarly, we now compare the effects of these variables for the progression-free survival prediction. Specifically, for pre-operative prediction at Setting 1, the significant variables are almost the same with the overall survival prediction, except here the biopsy histologic grade is also significant (HR=1.507; p=0.03). For post-operative prediction at Setting 1, the significant variables are also almost the same with the overall survival prediction, except that gross appearance is not significant. Most significant variables at Setting 1 remain significant at Setting 2, except that biopsy histologic grade and pN became insignificant. Interestingly, when combining only the significant variables as shown at Setting 3, the significant variables are CEA, the two imaging signatures, pT, pN, and chemotherapy. Comparing Setting 3 and Setting 4, the significant predictors remain the same in the two settings.

In all the four settings, the hazard ratios for chemotherapy treatment are lower than one, indicating the chemotherapy treatment reduces the risk of death (in other words, patients benefit from receiving the treatment with an increasing survival time). The two imaging signatures play a significant prediction role of survival in all the four settings. For pT and pN, at Setting 3, they are not significant for overall survival while significantly predictive of progression-free survival; at Setting 4, pN is a significant predictor for both overall and progression-free survival, while pT is only significantly predictive for progression-free survival.

## Discussion

In this study, we evaluated the combination of various clinical variables and quantitative CECT imaging descriptors for overall and progression-free survival prediction on gastric cancer patients. We identified five primary prognosis factors, including two pathological staging variables, the history of chemotherapy treatment, and two aggregated signatures from radiomics and deep learning. While multi-modal data have been increasingly used in machine learning modeling, our study provides a measurement on the quantitative effects of the examined multi-modal features for gastric cancer survival analysis. This can enhance gastric cancer patient prognostication.

We found that in the models with the highest c-indices, the two pathological staging variables, pT and pN, are correlated with survival with highest hazard ratios. This suggests that the pathological staging data including both the depth of mural invasion and nodal involvement are closely indicative of patient survival. It is noted that when combined with pre-operative variables (including the imaging signatures), the effects of pT and pN are dismissed for overall survival prediction. This may have two important indications. First, because of the lower c-index at Settings 3 and 4, we suspect this may have to do with the modeling method in the Cox model, where variables are simply linearly concatenated and thus may not be optimal to capture more complicated non-linear interactions when the aggregated imaging signatures are incorporated in the model. Additional work on developing advanced modeling methods is therefore warranted. Second, at Settings 3 and 4 we found that the deep learning signature maintains high hazard ratios (like at Settings 1 and 2), while pT and pN are insignificant. This implies that the proposed deep learning model can extract quantitative imaging features that have overlapping information with pT and pN for overall survival prediction. This is a finding that highlights the important utility of pre-operative CECT imaging data coupled with the proposed deep learning modeling techniques. Interestingly, when looking at the progression-free survival at Setting 3, both the two imaging signatures and pT and pN are significant predictors with a similar magnitude of hazard ratios, which indicates that the information in the pre-operative CECT imaging signatures and the information in the pathological staging markers are complementary to each other for the progression-free survival prediction. Such complementary effects may align with the observation that CECT images can visualize the invasion of tumor into gastric wall (T stage) and the enlarged regional lymph nodes (N stage). Finally, it is not surprising to see that post-operative chemotherapy, with a hazard ratio consistently lower than one, can significantly increase survival.

Radiomics are mathematically defined descriptors while deep learning features are less intuitive because of the complexity in deep neural networks. The two aggregated imaging signatures are identified as significant factors for both overall and progression-free survival prediction. These two signatures may convey distinct information on the high-dimensional CECT images. Radiomic features/signature quantify characteristics of the segmented intratumor regions. Tumor margins, or the peri-tumorous regions, may also carry active and predictive information related to patient outcomes (24). The deep learning signature derived specifically from the attention-guided VAE model can extract additional features from the approximate tumor regions (not necessarily limited to intra-tumor). In our analysis, when the pre- and post-operative data are combined, deep learning signature shows a higher hazard ratio (i.e., importance) than the radiomic signature for overall survival prediction, and a comparable hazard ratio for the progression-free survival prediction. This observation indicates that the radiomics features and deep learning-identified features play important yet different roles or interact distinctly in the two survival prediction tasks.

The focus of our study is to examine the effect and relationship of multi-modal features for gastric cancer survival prediction. Meanwhile, our model’s c-index values are in line with previously reported studies (3, 11). For example, a deep learning-based nomogram (11) achieved c-index of 0.802 and 0.792, respectively, for overall and disease-free survival of gastric cancer. A Cox proportional hazard model with the AJCC staging system showed c-index of 0.796 for overall survival on a gastric cancer cohort (3). Although these values cannot be directly compared due to the differences on study cohort, data modality, and evaluation setting, we put these numbers in the same context for a general overview of the survival prediction model’s performance. In addition, while these prediction models may not be directly used in their current capacities, the important findings of our study are the quantitative effects of the prognostic biomarkers identified from the multi-modality data, which can better inform clinicians for clinical decision-making. In particular, the pre-operative prediction of survival may provide early information to improve treatment planning and patient care.

Our study has some limitations. While our study included more than one thousand patients with complete data to enable the performed analyses, additional evaluation using external datasets will further validate our findings. The Cox model is more explainable but may be less effective to integrate non-linear interactions among multi-modal features. This study indicates the needs of developing more advanced models in future work. In addition, indications to chemotherapy were not consistently applied to the enrolled patients according to the NCCN guideline, which reflects a limitation of retrospective analysis. Finally, the tumor segmentation is semi-automated, which may have introduced certain level of dependence to the data annotators. While showing a high intra- and inter-observer agreement on segmentations, we expect to use fully automated and robust tumor segmentation methods when they become available.

## Conclusions

We integrated multi-modal data for gastric cancer survival prediction and evaluated their individual and combined effects. Our study showed that quantitative radiomics and deep learning imaging features are significant pre-operative predictors of survival, providing additional prognostic information to the pathological staging markers. Lower CEA levels and chemotherapy treatments independently increase survival chances. Our findings provide quantitative effect measures on these markers in pre- and post-operative survival prediction, which will enhance gastric cancer patient prognostication and benefit treatment planning

## Data Availability Statement

The datasets presented in this article are not currently available to the public because of internal regulations and considerations of our research and publishing plans. Readers who are interested to the datasets may send a request to wus3@upmc.edu.

## Ethics Statement

The studies involving human participants were reviewed and approved by an ethics committee of the First Affiliated Hospital with Nanjing Medical University (2018-SR-043) and the Institutional Review Board of University of Pittsburgh (STUDY19080135). The ethics committee waived the requirement of written informed consent for participation.

## Author Contributions

SW and YZ jointly conceived the concept and supervised the study. DH, YZ, and SW designed the methodology. DH implemented the models. DH and QL performed major data analysis. QL, YZ, and XL collected and pre-processed data and provided clinical expertise. LQ, QXF, and QL performed imaging data annotation and/or clinical data review/re-interpretation. DA contributed to model evaluation. All authors contributed to data analysis and result interpretation. DH, QL, YZ, and SW drafted the manuscript. All authors contributed to the article and approved the submitted version.

## Funding

This work was supported in part by an Amazon Machine Learning Research Award.

## Conflict of Interest

SW is a scientific consultant and stockholder of COGNISTX, Inc. SW has a research grant funded by Amazon.

The remaining authors declare that the research was conducted in the absence of any commercial or financial relationships that could be construed as a potential conflict of interest.

## Publisher’s Note

All claims expressed in this article are solely those of the authors and do not necessarily represent those of their affiliated organizations, or those of the publisher, the editors and the reviewers. Any product that may be evaluated in this article, or claim that may be made by its manufacturer, is not guaranteed or endorsed by the publisher.

## References

[1] Chen W Zheng R Baade PD Zhang S Zeng H Bray F. Cancer Statistics in China, 2015. CA: Cancer J Clin (2016) 66(2):115–32. doi: 10.3322/caac.21338 26808342

[2] Novotny AR Schuhmacher C Busch R Kattan MW Brennan MF Siewert JR. Predicting Individual Survival After Gastric Cancer Resection: Validation of a US-Derived Nomogram at a Single High-Volume Center in Europe. Ann Surg (2006) 243(1):74. doi: 10.1097/01.sla.0000194088.81126.85 16371739 PMC1449962

[3] Son T Sun J Choi S Cho M Kwon IG Kim HI. Multi-Institutional Validation of the 8th AJCC TNM Staging System for Gastric Cancer: Analysis of Survival Data From High-Volume Eastern Centers and the SEER Database. J Surg Oncol (2019) 120(4):676–84. doi: 10.1002/jso.25639 31338834

[4] In H Solsky I Palis B Langdon-Embry M Ajani J Sano T. Validation of the 8th Edition of the AJCC TNM Staging System for Gastric Cancer Using the National Cancer Database. Ann Surg Oncol (2017) 24(12):3683–91. doi: 10.1245/s10434-017-6078-x 28895113

[5] He X Wu W Lin Z Ding Y Si J Sun L-m. Validation of the American Joint Committee on Cancer (AJCC) Stage System for Gastric Cancer Patients: A Population-Based Analysis. Gastric Cancer (2018) 21(3):391–400. doi: 10.1007/s10120-017-0770-1 29052053

[6] Kono K Amemiya H Sekikawa T Iizuka H Takahashi A Fujii H. Clinicopathologic Features of Gastric Cancers Producing Alpha-Fetoprotein. Digestive Surg (2002) 19(5):359–65. doi: 10.1159/000065838 12435906

[7] Adachi Y Yasuda K Inomata M Sato K Shiraishi N Kitano S. Pathology and Prognosis of Gastric Carcinoma: Well Versus Poorly Differentiated Type. Cancer: Interdiscip Int J Am Cancer Soc (2000) 89(7):1418–24. doi: 10.1002/1097-0142(20001001)89:7<1418::AID-CNCR2>3.0.CO;2-A 11013353

[8] Hyung WJ Lee JH Choi SH Min JS Noh SH. Prognostic Impact of Lymphatic and/or Blood Vessel Invasion in Patients With Node-Negative Advanced Gastric Cancer. Ann Surg Oncol (2002) 9(6):562–7. doi: 10.1007/BF02573892 12095972

[9] Bilici A Seker M Ustaalioglu BBO Kefeli U Yildirim E Yavuzer D. Prognostic Significance of Perineural Invasion in Patients With Gastric Cancer Who Underwent Curative Resection. Ann Surg Oncol (2010) 17(8):2037–44. doi: 10.1245/s10434-010-1027-y 20333555

[10] Takahashi Y Takeuchi T Sakamoto J Touge T Mai M Ohkura H. The Usefulness of CEA and/or CA19-9 in Monitoring for Recurrence in Gastric Cancer Patients: A Prospective Clinical Study. Gastric Cancer (2003) 6(3):142–5. doi: 10.1007/s10120-003-0240-9 14520526

[11] Jiang Y Jin C Yu H Wu J Chen C Yuan Q. Development and Validation of a Deep Learning CT Signature to Predict Survival and Chemotherapy Benefit in Gastric Cancer: A Multicenter, Retrospective Study. Ann Surg (2020) 274(6):1153–61. doi: 10.1097/SLA.0000000000003778 31913871

[12] Jiang Y Chen C Xie J Wang W Zha X Lv W. Radiomics Signature of Computed Tomography Imaging for Prediction of Survival and Chemotherapeutic Benefits in Gastric Cancer. EBioMedicine (2018) 36:171–82. doi: 10.1016/j.ebiom.2018.09.007 PMC6197796 30224313

[13] LeCun Y Bengio Y Hinton G. Deep Learning. Nature (2015) 521(7553):436–44. doi: 10.1038/nature14539 26017442

[14] Mobadersany P Yousefi S Amgad M Gutman DA Barnholtz-Sloan JS Vega JEV. Predicting Cancer Outcomes From Histology and Genomics Using Convolutional Networks. Proc Natl Acad Sci (2018) 115(13):E2970–9. doi: 10.1073/pnas.1717139115 PMC5879673 29531073

[15] Ishwaran H Kogalur UB Blackstone EH Lauer MS. Random Survival Forests. Ann Appl Stat (2008) 2(3):841–60. doi: 10.1214/08-AOAS169

[16] Cox DR. Regression Models and Life-Tables. J R Stat Society: Ser B (Methodological) (1972) 34(2):187–202. doi: 10.1111/j.2517-6161.1972.tb00899.x

[17] Van Griethuysen JJ Fedorov A Parmar C Hosny A Aucoin N Narayan V. Computational Radiomics System to Decode the Radiographic Phenotype. Cancer Res (2017) 77(21):e104–e7. doi: 10.1158/0008-5472.CAN-17-0339 PMC5672828 29092951

[18] Breiman L. Random Forests. Mach Learn (2001) 45(1):5–32. doi: 10.1023/A:1010933404324

[19] Paszke A Gross S Massa F Lerer A Bradbury J Chanan G. eds. Pytorch: An Imperative Style, High-Performance Deep Learning Library. Advances in Neural Information Processing Systems. Vancouver, B.C., Canada: Curran Associates, Inc. (2019).

[20] Polsterl S Gupta P Wang L Conjeti S Katouzian A Navab N. Heterogeneous Ensembles for Predicting Survival of Metastatic, Castrate-Resistant Prostate Cancer Patients. F1000Res (2016) 5:2676. doi: 10.12688/f1000research.8231.1 28713544 PMC5500862

[21] Harrell FE Jr Lee KL Mark DB. Multivariable Prognostic Models: Issues in Developing Models, Evaluating Assumptions and Adequacy, and Measuring and Reducing Errors. Stat Med (1996) 15(4):361–87. doi: 10.1002/(SICI)1097-0258(19960229)15:4<361::AID-SIM168>3.0.CO;2-4 8668867

[22] Dekking FM Kraaikamp C Lopuhaä HP Meester LE. A Modern Introduction to Probability and Statistics: Understanding Why and How. Springer Science & Business Media (2005).

[23] Ajani J D’Amico T Bentrem D Chao J Corvera C Das P. NCCN Clinical Practice Guidelines in Oncology: Gastric Cancer. National Comprehensive Cancer Network (2015).

[24] Chen T Xu L Dong X Li Y Yu J Xiong W. The Roles of CT and EUS in the Preoperative Evaluation of Gastric Gastrointestinal Stromal Tumors Larger Than 2 Cm. Eur Radiol (2019) 29(5):2481–9. doi: 10.1007/s00330-018-5945-6 30617491

