# Supplementary material for: Identifying Prognostic Markers From Clinical, Radiomics, and Deep Learning Imaging Features for Gastric Cancer Survival Prediction
Source: Front Oncol. 2022 Feb 2;11:725889. doi: 10.3389/fonc.2021.725889 (PMC8847133; doi:10.3389/fonc.2021.725889)
Supplement: Supplementary file 1 [file DataSheet_1.docx]

Identifying prognostic markers from clinical, radiomics, and deep learning imaging features for gastric cancer survival prediction

# Supplemental Text

## CT imaging parameters

The detailed CT imaging parameters and protocols were summarized as following. All CECT imaging examinations were performed with a 64-MDCT (SOMATOM, Definition AS+, Siemens, Forchheim, Germany) in a single institution before the surgical treatments. Key CT imaging parameters were: 120 kVp; effective 180 mAs; rotation time, 0.5 s; detector collimation, 32×1.2 mm; matrix, 512 × 512; reconstruction section thickness, 1.5 mm, and filter back projection for reconstruction. Non-ionic contrast medium (Ultravist; 300 mgI/mL, Bayer Schering Pharma AG, Berlin, Germany) was injected into the antecubital vein with a dose of 60-110 ml (1.5 ml per kilogram of body weight) and an average injection rate of 3.0 ml s–1 through a venous indwelling needle (20 or 22 gauge). By using the automated scan-triggering software (Care-Bolus; Siemens Medical Systems, Iselin, NJ), the arterial phase and portal venous phase scan started automatically with a 15 second delay and a 50 second delay after the attenuation value of abdominal aorta reached 100 HU, respectively. After the acquisition of the portal venous phase images, delayed phase images were acquired with a delay of 180 seconds.

## Measurement details on clinical variables

The qualitative radiologic staging variables, rT and rN, were retrospectively reinterpreted by two experienced radiologists (CL and LQ). Both readers were members of the data-acquisition institution’s gastrointestinal disease management team with 3-year and 10-year experience in abdominal imaging and had read > 1, 000 gastric CT scans by the time of this study. To minimize potential bias, the two radiologists interpreted all cases independently and for the cases that their staging were not concordant, they together reviewed and discussed case by case and reached a consensus. During the interpretation, the two radiologists were blinded to clinicopathological characteristics except the endoscopically proven locations of the tumors. The rT stage was classified as rT1, rT2, rT3, and rT4, which referred to the standards of Kim JW et al 12 and Ahn HS et al 13. A patient’s metastatic lymph node on CT imaging was defined as lymph node with short-axis diameter > 10 mm, ratio of shortest to longest axes > 0.7, marked heterogeneous enhancement, central necrosis, clustering. No regional LN+, 1-2 LN+, 3-6 LN+, 7-15 LN+, 7-15 LN+ and > 15 LN+ were defined as N0, N1, N2, N3 and N4 stage, respectively. The pre-operative histologic grades at endoscopic biopsy are evaluated according to the WHO Classification of Tumors of the Digestive System 14. The histopathological specimens are analyzed by certified pathologists with subspecialty training in gastrointestinal pathology, where the pathologists are blinded to imaging findings. LVI is defined as the presence of malignant cells within blood vessels and/or lymphatics. PNI is defined as the infiltration of carcinoma cells into the perineurium or neural fascicles. Pathological stages are assessed according to the eighth edition AJCC Cancer Staging Manual.
